# Different efficacy of tyrosine kinase inhibitors by *KIT* and *PGFRA* mutations identified in circulating tumor DNA for the treatment of refractory gastrointestinal stromal tumors

**DOI:** 10.1038/s44276-024-00073-7

**Published:** 2024-07-25

**Authors:** Tadayoshi Hashimoto, Yoshiaki Nakamura, Yoshito Komatsu, Satoshi Yuki, Naoki Takahashi, Naohiro Okano, Hidekazu Hirano, Koushiro Ohtsubo, Takashi Ohta, Eiji Oki, Tomohiro Nishina, Hisateru Yasui, Hisato Kawakami, Taito Esaki, Nozomu Machida, Ayako Doi, Shogen Boku, Toshihiro Kudo, Yoshiyuki Yamamoto, Akiyoshi Kanazawa, Tadamichi Denda, Masahiro Goto, Naoko Iida, Hiroshi Ozaki, Taro Shibuki, Mitsuho Imai, Takao Fujisawa, Hideaki Bando, Yoichi Naito, Takayuki Yoshino

**Affiliations:** 1 Translational Research Support Office, National Cancer Center Hospital East, Kashiwa, Japan; 2 Department of Gastroenterology and Gastrointestinal Oncology, National Cancer Center Hospital East, Kashiwa, Japan; 3 Department of Cancer Center, Hokkaido University Hospital, Sapporo, Japan; 4 Department of Gastroenterology and Hepatology, Hokkaido University Hospital, Sapporo, Japan; 5 Department of Gastroenterology, Saitama Cancer Center, Saitama, Japan; 6 Department of Medical Oncology, Kyorin University Faculty of Medicine, Tokyo, Japan; 7 Department of Gastrointestinal Medical Oncology, National Cancer Center Hospital, Tsukiji, Japan; 8 Department of Medical Oncology, Cancer Research Institute, Kanazawa University, Kanazawa, Japan; 9 Department of Clinical Oncology, Kansai Rosai Hospital, Hyogo, Japan; 10 Department of Surgery and Science, Graduate School of Medical Sciences, Kyushu University, Fukuoka, Japan; 11 Department of Gastrointestinal Medical Oncology, National Hospital Organization Shikoku Cancer Center, Matsuyama, Japan; 12 Department of Medical Oncology, Kobe City Medical Center General Hospital, Hyogo, Japan; 13 Department of Medical Oncology, Kindai University Hospital, Osaka, Japan; 14 Department of Gastrointestinal and Medical oncology, National Hospital Organization Kyushu Cancer Center, Fukuoka, Japan; 15 Department of Gastroenterology, Kanagawa Cancer Center, Yokohama, Japan; 16 Department of Clinical Oncology, St. Marianna University School of Medicine, Kanagawa, Japan; 17 Cancer Treatment Center, Kansai Medical University, Osaka, Japan; 18 Department of Medical Oncology, Osaka International Cancer Institute Osaka Prefectural Hospital Organization, Osaka, Japan; 19 Department of Gastroenterology, University of Tsukuba Hospital, Ibaraki, Japan; 20 Department of Surgery Shimane Prefectural Central Hospital, Shimane, Japan; 21 Division of Gastroenterology, Chiba Cancer Center, Chiba, Japan; 22 Cancer Chemotherapy Center, Osaka Medical and Pharmaceutical University Hospital, Takatsuki, Japan; 23 Department of Head and Neck Medical Oncology, National Cancer Center Hospital East, Kashiwa, Japan; 24 Department of Medical Oncology, National Cancer Center Hospital East, Kashiwa, Japan

## Abstract

**Background:**

While advanced gastrointestinal stromal tumors (GISTs) are primarily treated with tyrosine kinase inhibitors (TKIs), acquired resistance from specific mutations in *KIT* or *PDGFRA* frequently occurs. We aimed to assess the utility of circulating tumor DNA (ctDNA) as a modality of therapeutic decision-making in advanced GIST.

**Methods:**

We conducted a pooled analysis of SCRUM-Japan studies for advanced GIST patients. We compared patient characteristics analyzed with tissue and blood samples, assessed gene alteration profiles, and evaluated prognostic implications from ctDNA status.

**Results:**

In 133 patients, tissue and blood samples were analyzed for 89 and 44 patients, respectively. ctDNA was detected in 72.7% of cases; no prior treatment or progressive disease was significantly associated with ctDNA-positivity. ctDNA-positive patients had significantly shorter progression-free survival compared with ctDNA-negative patients (hazard ratio = 3.92; *P* = 0.007). ctDNA genotyping revealed a complex landscape of gene alterations, characterized by multi-exonic mutations in *KIT*, compared with tissue-based analysis. Patients who received TKIs matched to the identified *KIT* mutation in ctDNA demonstrated significantly longer PFS than those with unmatched treatment (median, 8.23 vs. 2.43 months; *P* < 0.001).

**Conclusions:**

ctDNA-based analysis facilitates assessment of disease status and genomic profiles, thus potentially assisting in identifying optimal therapeutic strategies for advanced GIST patients.

## Introduction

Gastrointestinal stromal tumors (GISTs), arising from the interstitial cells of Cajal, represent a rare but predominant soft-tissue sarcoma within the gastrointestinal tract [1]. The main causative events for GIST involve gain-of-function mutations in the *KIT* or *PDGFRA* genes, encoding receptor tyrosine kinases, that are present in approximately 70% and 15% of cases, respectively [2, 3]. In the remaining 10% to 15% of patients, other driver genes such as *BRAF*, *NF1*, and *SDH* are implicated; some patients also lack specific genetic mutations [4].

The development of imatinib, a small molecule that inhibits the activity of the KIT tyrosine kinase, has brought about a remarkable paradigm shift in the clinical management of cancer patients. Other tyrosine kinase inhibitors (TKIs) such as sunitinib and regorafenib are current standard-of-care treatments [5, 6]. However, the majority of patients that undergo imatinib therapy commonly experience drug resistance, known as secondary resistance [7, 8]. The primary cause of secondary resistance (approximately 70% of cases) is secondary mutations in *KIT* or *PDGFRA*, specifically in the ATP-binding pocket encoded by exons 13 and 14 or in the activation loop encoded by exons 17 and 18 [9–12]. The distinct therapeutic effects by TKIs can be attributed to the differences in the exons harboring these mutations. While clinical guidelines provide recommendations concerning the sequence of TKI treatment, a therapeutic strategy targeting secondary gene mutations has not yet been established [13].

There has been a recent surge of interest in the investigation of circulating tumor DNA (ctDNA) as a less-invasive liquid biopsy technique compared with tissue sampling [14–16]. Longitudinal ctDNA testing may allow the detection of acquired resistance variants generated from selective pressure from kinase inhibitors. While this method may aid in the best selection of the next line of therapy determined by the acquired resistance genotype in GIST patients, its utility has not been elucidated [17, 18].

The present study aimed to identify gene alteration profiles in GIST using data derived from tissue and blood samples separately, evaluate the impact of ctDNA on prognostication, and identify resistance-associated alterations in patients with advanced GIST. In pursuit of these objectives, we integrated the clinical trials on the SCRUM-Japan platform, encompassing GI-SCREEN, GOZILA, MOSTAR-SCREEN, and MONSTAR-SCREE-2, which are nationwide molecular profiling programs in Japan [19, 20].

## Patients & methods

### Patients

This study included patients with advanced GIST who participated in SURUM-Japan trials, including GI-SCREEN (UMIN000016344), GOZILA (UMIN000029315), MONSTAR-SCREEN (UMIN000036749), and MONSTAR-SCREEN-2 (UMIN000043899). The study protocol was approved by the Institutional Review Board of National Cancer Center Hospital East (UMIN000049334).

GI-SCREEN included patients with advanced gastrointestinal cancers between February 2015 and April 2019, and tumor tissue samples were sequenced. GOZILA is a plasma ctDNA profiling study initiated in January 2018. Patients with advanced gastrointestinal cancers who had disease progression during systemic chemotherapy or targeted therapy were enrolled, and plasma ctDNA was sequenced. MONSTAR-SCREEN enrolled patients with advanced solid tumors between May 2019 and February 2021 and performed longitudinal plasma ctDNA genotyping before and after systemic therapy. MONSTAR-SCREEN-2 was launched in May 2021, and whole-exome and transcriptome analyses of tumor tissue and plasma have been conducted in patients with advanced solid tumors.

### Analysis of tissue samples

In GI-SCREEN, archival formalin-fixed paraffin-embedded (FFPE) tumor tissues were collected and analyzed between February 2015 and March 2017 using the Oncomine Comprehensive Assay v1 (Thermo Fisher Scientific, Inc., Waltham, MA), a multi-biomarker next-generation sequencing (NGS) assay that covers 143 of the most relevant cancer genes. The Oncomine Comprehensive Assay v3 covering 161 of the most relevant cancer genes was used between April 2017 and April 2019. These assays identify relevant single nucleotide variants (SNVs), copy number variations, gene fusions, and indels in a streamlined workflow. MONSTAR-SCREEN-2 uses the CARIS MI Profile assay (CARIS Life Sciences, Phoenix, AZ), a whole-exome and whole-transcriptome sequencing assay of tumor tissue samples. This technology includes whole-exome sequencing for DNA mutations, copy number alterations, insertions/deletions, and genomic signatures for loss of heterogeneity, microsatellite instability (MSI), and tumor mutational burden (TMB). The targeted gene panels in these assays include *KIT* and *PDGFRA*.

### Analysis of blood samples

In GOZILA, plasma ctDNA was analyzed using Guardant360 (Guardant Health, Inc., Redwood City, CA.), a comprehensive ctDNA sequencing encompassing 74 genes. MONSTAR-SCREEN profiled ctDNA using FoundationOne Liquid CDx (Foundation Medicine, Inc., Cambridge, MA), which targets alterations of 324 genes and complex biomarkers, such as MSI, TMB, and tumor fraction. The targeted genes in these assays include *KIT* and *PDGFRA*. Somatic cell-free DNA alterations were identified using a proprietary bioinformatics pipeline. ctDNA fraction was measured by the maximum variant allelic fraction (VAF). According to the assay’s technical information, the detection sensitivity for genomic alterations is delineated as follows: In FoundationOne Liquid CDx, for short genomic variants, the assay exhibits median Limits of Detection (LOD) of 0.82% and 0.40% VAF for standard and enhanced sensitivity, respectively. In contrast, the Guardant360 assay demonstrates established LODs ranging from 1.0% to 1.5% mutant allele frequency with a 5 ng cell-free DNA input and 0.2% mutant allele frequency with a 30 ng input. To adjust and estimate the clonality for somatic SNVs and indels, relative clonality was initially defined as alteration VAF divided by maximum somatic VAF in the sample. We then conducted a comparison of the ctDNA clonality of *KIT* or *PDGFRA* mutations by relative clonality.

### Statistical analysis

Clinical characteristics were compared using the Chi-squared test for categorical variables and the Mann–Whitney U test for continuous variables. Patients were classified into three groups on the basis of disease status at the time of enrollment: “no prior targeted therapy”, “progressive disease” (which was characterized by a change in treatment within one month following enrollment), and “on treatment” (in which treatment was continued for more than one month post-enrollment). Regarding the therapeutic spectrum of TKIs for *KIT* or *PDGFRA* mutational status in this study, we defined imatinib as effective for *KIT* exon 11 and *PDGFRA* exon 18 (other than D842V) mutations, sunitinib as effective for *KIT* exon 9/11/13/14 mutations, and regorafenib as effective for *KIT* exon 9/13 (other than V654A)/14/17/18 mutations, as suggested by previous reports [21, 22]. We summarized the associations between the mutational status and the efficacy of different TKIs in the present study (Supplementary Fig. 1). Overall survival (OS) was estimated as the time from the date of enrollment to the date of death from any cause. Progression-free survival (PFS) was estimated for each treatment after enrollment and defined as the time from the date of treatment initiation to either the date of disease progression or death from any cause. OS and PFS were calculated using the Kaplan–Meier method and assessed using the log-rank test. Cox proportional hazards models were employed for both univariate and multivariate analyses. *P*-values of <0.05 were considered statistically significant. Statistical analyses were performed using the R Statistics software program, version 4.2.3. The data cut-off date for the analyses was June 30, 2023.

## Results

### Patient characteristics associated with tissue genotyping failure and ctDNA detection

A total of 133 patients with advanced GIST were included in SCRUM-Japan studies, including 79 of 5743 enrolled in GI-SCREEN, 36 of 5499 enrolled in GOZILA, 8 of 2224 in MONSTAR-SCREEN, and 10 of 1897 enrolled in MONSTAR-SCREEN-2 (Supplementary Fig. 2). Tissue genotyping was not performed in seven patients because of insufficient sample quality. Of the remaining 126 patients, 82 and 44 patients underwent tissue and ctDNA genotyping, respectively.

The baseline characteristics of patients examined by tissue genotyping are shown in Table 1. Among the 82 patients who underwent tissue genotyping, 65 (79.3%) had successfully sequenced results, whereas sequencing failed in 17 (20.7%). Specimens collected from a metastatic site and prior treatment were significantly associated with tissue genotyping failure (*P* = 0.047 and *P* = 0.033, respectively).

**Table 1:** Baseline characteristics of patients analyzed with tissue samples.

Characteristics	Success (*N* = 65)	Failure (*N* = 17)	*P* value
Age (years)			
>65	32 (49.2%)	10 (58.8%)	1.00
≤65	33 (50.8%)	7 (41.2%)	
Sex			
Male	37 (56.9%)	9 (52.9%)	1.00
Female	28 (43.1%)	8 (47.1%)	
Primary site of tumor			
Stomach	26 (40.0%)	9 (52.9%)	0.112
Small intestine	31 (47.4%)	5 (29.4%)	
Rectum	3 (4.6%)	3(17.6%)	
Others	5 (7.7%)	0	
Specimen collection methods			
Biopsy	9 (13.8%)	5 (29.4%)	0.234
Surgical resection	53 (81.5%)	12 (70.6%)	
Others	3 (4.6%)	0	
Specimen collection sites			
Primary lesion	46 (70.8%)	7 (41.2%)	0.047
Metastatic lesion	19 (29.2%)	10 (58.8%)	
Liver	11	6	
Peritoneum	6	3	
Lung	1	0	
Bone	0	1	
Skin	1	0	
Prior targeted therapy			
Performed	12 (18.5%)	8 (47.1%)	0.033
Not performed	53 (81.5%)	9 (52.9%)	

The baseline characteristics of the 44 patients who underwent ctDNA analysis are shown in Table 2. ctDNA was not detected in 12 (27.3%) patients. Patients on treatment were not likely to have detectable ctDNA compared with patients with those on progressive disease or those with no prior targeted therapy at the time of blood sampling (*P* = 0.027). The median duration from ctDNA analysis to the subsequent manifestation of clinical disease status was 8.0 days (95% confidence interval, 2.0–14.5) for patients classified as progressive disease and 127.5 days (95% confidence interval, 42.5–287.5) for those classified as on treat, respectively. Notably, factors such as tumor location, presence of primary tumor, metastatic organs, and the number of metastatic organs were not associated with ctDNA positivity.

**Table 2:** Comparison of patients’ characteristics according to ctDNA status at the blood sampling.

Characteristics		ctDNA positive	ctDNA negative	*P* value
		(*N* = 32)	(*N* = 12)	
Age, years				
	>65	13 (40.6%)	5 (41.7%)	1.00
	≤65	19 (59.45%)	7 (58.3%)	
Sex				
	Male	17 (53.1%)	4 (33.3%)	0.406
	Female	15 (46.9%)	8 (66.7%)	
Primary site of tumor				
	Stomach	8 (25.0%)	5 (41.7%)	0.630
	Small intestine	19 (59.4%)	4 (33.3%)	
	Rectum	2 (6.2%)	1 (8.3%)	
	Others	3 (9.4%)	2 (16.7%)	
Resection of primary tumor				
	Performed	21 (65.6%)	8 (66.7%)	1.00
	Not performed	11 (34.4%)	4 (33.3%)	
Number of metastatic organ sites				
	≤1	27 (84.4%)	10 (83.3%)	1.00
	≥2	5 (15.6%)	2 (16.7%)	
Metastatic organs^a^				
	Liver	18 (56.2%)	6 (50.0%)	0.910
	Peritoneum	10 (31.2%)	6 (50.0%)	0.385
	Lung	4 (12.5%)	1 (8.3%)	1.00
	Lymph nodes	1 (3.1%)	2 (16.7%)	0.344
	Others	3 (9.4%)	0	0.685
Number of previous treatment regimens				
	≤2	23 (71.9%)	10 (83.3%)	0.734
	≥3	9 (28.1%)	2 (16.7%)	
Disease status				
	No prior targeted therapy	5 (15.6%)	0	0.027
	Progressive disease status	24 (75.0%)	7 (58.3%)	
	On treatment	3 (9.4%)	5 (41.7%)	

*ctDNA* circulating tumor DNA.

^a^Duplications are included.

### Survival analysis in accordance with ctDNA status

**Fig. 1:**
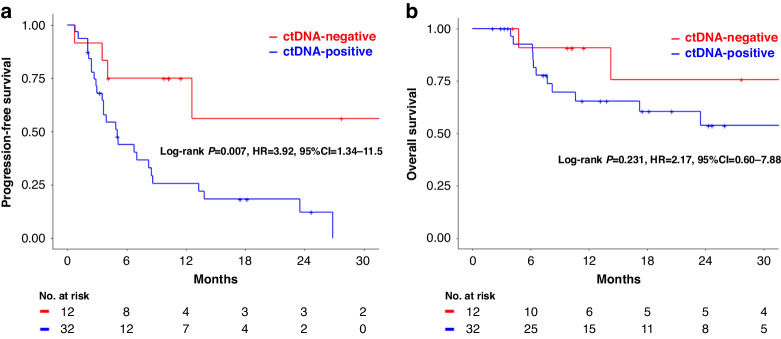
Survival analysis in accordance with ctDNA status. **a** Progression-free survival in accordance with ctDNA status: PFS was significantly shorter in patients with ctDNA-positive compared to those with ctDNA-negative. **b** Overall survival in accordance with ctDNA status: patients with ctDNA-positive exhibited a trend towards less favourable OS compared to those with ctDNA-negative.

We evaluated PFS and OS in accordance with ctDNA status. PFS was significantly worse in the ctDNA-positive group compared with the ctDNA-negative group (HR, 3.92; 95% CI, 1.34–11.5; log-rank *P* = 0.007) (Fig. 1a). OS in the ctDNA-positive group tended to be less favorable compared with the ctDNA-negative group, but without statistical significance (HR 2.17; 95% CI 0.60–7.88; log-rank *P* = 0.231) (Fig. 1b). Cox multivariate analysis, integrating potential confounding variables, demonstrated that ctDNA negativity was an independent prognostic factor for PFS (HR 3.46; 95% CI 1.07–11.2; *P* = 0.038) (Supplementary Table 1). Furthermore, we analyzed PFS, focusing on 39 patients clinically determined to be PD (PD and on treatment statuses) by physicians after treatment. Consequently, PFS was significantly longer in ctDNA-negative patients (HR 3.15; 95% CI 1.06–9.31; log-rank *P* = 0.038), consistent with the overall sample analysis (Supplementary Fig. 3).

### Landscape of genomic alterations identified by tissue and ctDNA genotyping

**Fig. 2:**
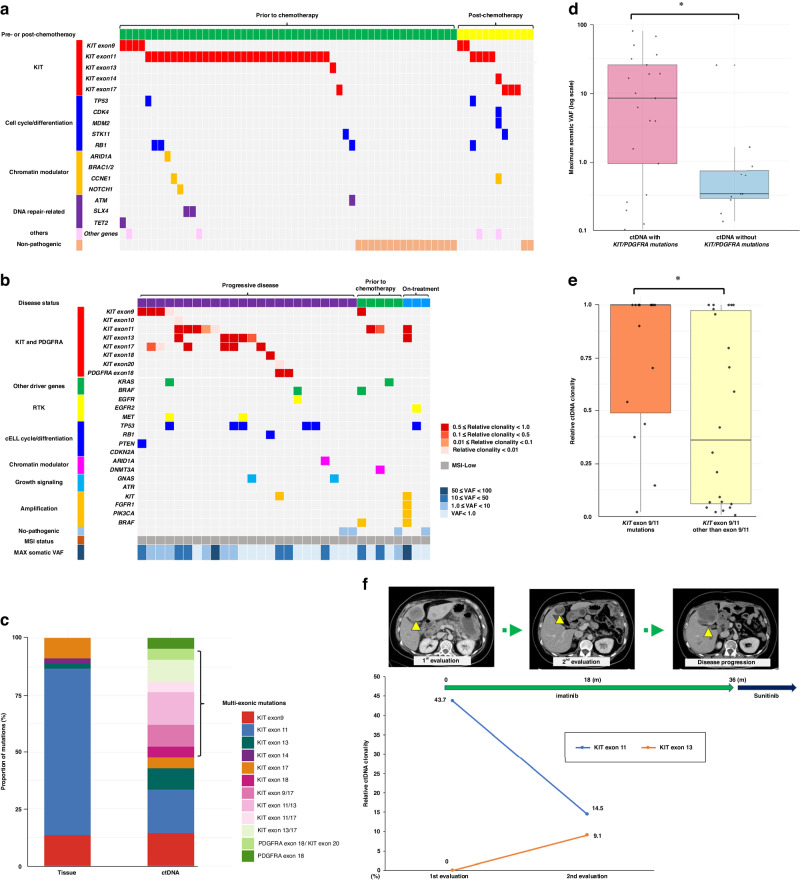
Genomic alterations identified by tissue and ctDNA genotyping. **a** Landscape of genomic alterations identified with tissue samples in patients with advanced GIST: mutation profiles in tissue samples are depicted separately for pre- and post-chemotherapy timepoints. Specific mutations in *KIT* exons are highlighted, with other alterations categorised by gene function, including cell cycle and differentiation, chromatin modulation, DNA repair-related genes, and others. **b** Landscape of genomic alterations identified with ctDNA analysis in patients with advanced GIST: genomic alterations identified in ctDNA are grouped into three stages: progressive disease, prior to chemotherapy, and on treatment. The maximum somatic variant allele frequency for each patient is depicted at the bottom. Mutations in *KIT* and *PDGFRA* are illustrated with a gradient representing relative clonality. Alterations in genes other than *KIT* and *PDGFRA* are categorised by function into driver genes, receptor tyrosine kinases, cell cycle and differentiation, chromatin modulators, and growth signalling pathways. Gene amplifications are marked in orange. **c** Comparison between tissue and ctDNA genotyping regarding *KIT* and *PDGFRA* mutations: the proportion of various mutation patterns in *KIT* and *PDGFRA* are shown cumulatively for both tissue genotyping and ctDNA genotyping. **d** Comparison between maximum somatic VAF of ctDNA with and without *KIT* and *PDGFRA* mutations: ctDNA harbouring *KIT* or *PDGFRA* mutations exhibits a significantly higher maximum somatic VAF compared to samples without these mutations. **e** Relative ctDNA clonality of *KIT* and *PDGFRA* mutations: *KIT* mutations other than exons 9/11 demonstrate significantly lower ctDNA clonality compared to mutations in *KIT* exons 9/11. **f** Dynamic change of ctDNA clonality of *KIT* and *PDGFRA* mutations in Patient #1: upper CT images illustrate the dynamic response of liver metastases to treatment, while the line graph below shows the corresponding changes in relative ctDNA clonality.

We examined the landscape of genomic alterations identified through tissue genotyping (Fig. 2a). The presence of *KIT* exon 11 mutation (50.8%) was observed, followed by mutations in *KIT* exons 9 (9.2%) and 17 (6.2%). Notably, each of these mutations occurred as sole exon mutations. Among other pathogenic mutations, mutations in genes related to the cell cycle or cell differentiation pathways (12.3%), particularly *RB1* mutations (6.2%), were frequently observed.

ctDNA genotyping, performed in the 32 patients with ctDNA positivity, revealed a different *KIT* mutational pattern from that obtained with tissue-based genotyping (Fig. 2b). Sole *KIT* exon 9 (9.4%) or 11 (15.6%) mutations were less prevalent; instead, multi-exonic mutations in *KIT* encompassing exons 9, 10, 11, 13, 17, 18, and 20 were more prevalent (25.0%). Among other pathogenic mutations, *TP53* mutations were most frequently observed, accounting for 18.8% of patients. No patients exhibited high microsatellite instability. In cases where mutations in genes other than *KIT* display high VAF, resulting in a relatively lower VAF for the *KIT* gene mutation, this would consequently lead to a higher maximum VAF and a proportionately lower clonality for the *KIT* mutation. This phenomenon is illustrated in row 9, which presents a *KIT* exon 11 mutation with a VAF of 0.36% and an *STK11* mutation with a maximum somatic VAF of 50.1%, yielding a calculated relative clonality of 0.007 for the *KIT* mutation. We next compared *KIT* and *PDGFRA* alterations between tissue and blood genotyping results (Fig. 2c). The proportion of *KIT* and *PDGFRA* mutations in tissue and blood samples was similar, comprising 69.2% in tissue (45/65) and 68.8% in blood (22/32). However, blood samples exhibited a more diverse spectrum of *KIT* mutations, particularly evident in exon 11/13 (0% vs. 14.3%, *P* < 0.01) and exon 13/17 (0% vs. 9.5%, *P* < 0.01).

### ctDNA clonality and the dynamic change of *KIT* and *PDGFRA* mutations

To elucidate the ctDNA clonality patterns related to *KIT* and *PDGFRA* mutations, as well as other alterations, we compared the maximum somatic VAF of ctDNA carrying *KIT* or *PDGFRA* mutations against those harboring other mutations (Fig. 2d). The maximum somatic VAF of ctDNA with *KIT* or *PDGFRA* mutations was significantly higher than that without these mutations (median: 8.36 vs. 0.33, *P* = 0.009). We next compared the ctDNA clonality of *KIT* exon 9/11 mutations and *KIT* mutations other than exon 9/11 (Fig. 2e). The median clonality of *KIT* exon 9/11 mutations was 100% (range, 2.0–100), while the *KIT* mutations other than exon 9/11 had a significantly lower median ctDNA clonality of 36.0% (range, 0.6–100) (*P* = 0.044).

To investigate the dynamic change in ctDNA alterations throughout TKI therapy, we focused on two patients who underwent repeated ctDNA assessment and had detectable *KIT* or *PDGFRA*. Details of the pathogenic mutation status from multiple blood samplings for three patients are provided in Supplementary Table 2. Patient #1 had advanced GIST with multiple liver metastases and received partial gastrectomy for peritonitis because of perforation of the primary lesion. ctDNA testing was performed prior to imatinib and 17.9 months after initiation of imatinib. Compared with the pretreatment result, the clonality of the *KIT* exon 11 mutation decreased after imatinib treatment from 43.7% to 14.5%, whereas *KIT* exon 13 mutation newly emerged with a clonality of 9.1%. The disease remained stable at 12 months from the second ctDNA evaluation but progressed markedly after 18 months; the patient was switched to sunitinib treatment, which is currently ongoing (Fig. 2f). Patient #2 underwent an initial ctDNA test before the initiation of imatinib and a second ctDNA test after disease progression on imatinib with an interval of 2.4 months. In the case of patient #2, avapritinib, which is the preferred therapeutic option for treating GIST harboring the *PDGFRA* D842V mutation in Western countries, has not yet received regulatory approval in our healthcare system. Consequently, imatinib is commonly employed as the standard treatment, irrespective of the underlying mutational profile. Furthermore, the specific alteration identified in this patient was D842_S847 > EF, a unique molecular event that is distinct from the well-characterized non-D842V mutations. Given these considerations, the decision was made to initiate treatment with imatinib, in line with the prevailing clinical practice in our country. The clonality of *PDGFRA* exon 18 mutation increased along with disease progression, and a novel mutation within *KIT* exon 20 emerged (Supplementary Fig. 4).

### The relationships between *KIT* or *PDGFRA* mutation status and prior and subsequent TKIs in patients with ctDNA positivity

**Fig. 3:**
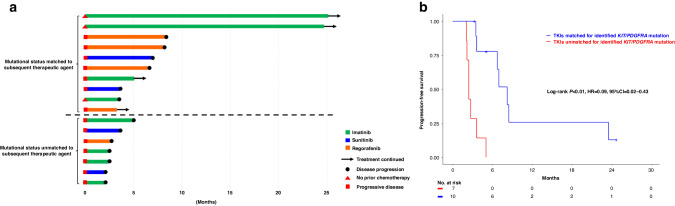
The efficacy of subsequent TKIs in accordance with their mutational status. **a** Swimmer plot of treatment duration and clinical outcome in accordance with mutational status matched or unmatched to subsequent therapy: swimmer plot depicted treatment duration and clinical outcomes for patients receiving TKIs matched to their mutational status (top section) and those treated with non-matched TKIs (bottom section). Disease status at treatment initiation, therapeutic agents used, and continuity of treatment are indicated for each patient. **b** Progression-free survival in accordance with the suitability of TKIs for *KIT* or *PDGFRA* mutations: patients treated with TKIs matched to their specific *KIT* or *PDGFRA* mutation profiles exhibited significantly longer PFS compared to those who received mismatched TKIs.

Out of the 35 samples with ctDNA positivity, which included 3 patients with multiple samples, 18 samples had *KIT* or *PDGFRA* mutations at progressive disease status. We evaluated the association between the efficacy of preceding TKIs and *KIT* or *PDGFRA* mutational status (Table 3). Within this sample subset, 14 (77.8%) harbored *KIT* exon mutations resistant to the preceding TKIs. Furthermore, we assessed the efficacy of subsequent TKIs for 17 patients harboring *KIT* or *PDGFRA* mutations in accordance with their mutational status (Fig. 3a). Patients who were subsequently treated with TKIs that matched their mutational status had a significantly longer PFS compared with those who received TKIs that were not matched [median PFS, 8.23 vs. 2.43 months; hazard ratio (HR), 0.09; 95% confidence interval, 0.02–0.43; log-rank *P* < 0.001] (Fig. 3b).

**Table 3:** The relationship between *KIT or PDGFRA* mutation status and preceding TKIs.

Sample No.	Preceding TKIs	Mutational status^a^	*KIT*							*PDGFRA*
			Exon 9	Exon 10	Exon 11	Exon 13	Exon 17	Exon 18	Exon 20	Exon 18
1	IM	Resistant	A502_Y503dup	–	–	–	N822K N822Y	–	–	–
2	SU	No-resistant	A502_Y503dup	–	–	–	–	–	–	–
3	SU	Resistant	–	–	–	–	D820Y	–	–	–
4	REG	Resistant	–	–	W557G	–	–	–	–	–
5	REG	Resistant	–	–	–	V654A	–	–	–	–
6	SU	No-resistant	–	–	–	V654A	–	–	–	–
7	SU	Resistant	–	–	V569_L576del		N822K	–	–	–
8	SU	Resistant	–	–	–	S628G K642E	N822K	–	–	–
9	IM	Resistant	–	–	–	–	–	A829P	–	–
10	REG	Resistant	–	–	–	V654A	N822K	–	–	–
11	IM	No-resistant	–	–	W557_E561del	–	–	–	–	–
12	SU	Resistant	–	F529S	E554_K558del	V654A	–	–	–	–
13	IM	Resistant	A502_Y503dup	–	–	–	–	–	–	–
14	REG	Resistant	–	–		–	–	–	E925K	D842_S847 > EF
15	IM	Resistant	A502_Y503dup	–		–	D820E	–	–	–
16	IM	No-resistant	–	–	D579del	–	–	–	–	–
17-1	REG	Resistant	–	–	–	–	–	–	–	D842_S847 > EF
17-2	IM	Resistant	–	–	–	–	–	–	D901N E925K	D842_S847 > EF

*TKIs* tyrosine kinase inhibitors, *IM* imatinib, *SU* sunitinib, *REG* regorafenib, *PD* progression disease, *dup* duplication, *del* deletion, *>* deletion-insertion.

^a^KIT and PDGFRA mutation status to the most prior tyrosine kinase inhibitor was judged according to the previous reports refs. [23, 24]

## Discussion

In the present study, we performed comprehensive genomic profiling of advanced GIST using the SCRUM-Japan platform. Our findings suggest that genomic characterization via liquid biopsies offers an enhanced understanding of the genetic diversity, heterogeneity, and dynamic genomic changes in GIST throughout therapeutic intervention compared with tissue-based analyses. Importantly, patients administered TKIs specifically tailored to mutations identified through ctDNA genotyping demonstrated markedly improved therapeutic outcomes relative to those receiving non-tailored TKIs. Due to the small sample size and potential background biases, caution should be exercised when interpreting these results. Further validation in larger cohorts is needed to confirm the clinical utility of this approach. Additionally, we discerned a robust association between ctDNA-positivity and progressive disease status, indicating ctDNA-positivity as a pivotal prognostic factor. These observations imply that ctDNA-based analysis can delineate intricate genetic profiles, which reflect not only the therapeutic lineage but also the contemporaneous disease status and help guide the establishment of optimal treatment strategies for patients with advanced GIST.

The success rate for tissue sample analysis in this study was approximately 80%. The collection of specimens from metastatic lesions and prior treatment were significant risk factors for analysis failure. These samples chiefly comprised archival tissues, and a success rate lower than expected may be from an insufficient quantity of samples because of minuscule metastatic lesions or compromised DNA quality. This is consistent with a previous report suggesting the importance of DNA quality and integrity for achieving successful sequencing [23]. Conversely, the ctDNA positivity of 72.7% in this study (including 36 and 8 patients evaluated with Guardant 360 and FoundationOne Liquid CDx, respectively) was superior to the 45–56.3% reported in retrospective studies [24, 25] and comparable to the 77.3% observed in the phase III INTRIGUE trial using Guardant 360 [18], but inferior to the 86.3% reported in the phase III VOYAGER trial [17], where Guardant 360 was used for ctDNA assessment in patients with heavily treated advanced GIST. These observations suggest that ctDNA positivity is contingent upon the tumor burden at the time of blood sampling. Furthermore, our investigation indicates that disease status at the time of sampling is significantly associated with ctDNA positivity, mirroring previous reports that showed that localized disease or prior treatment was linked to ctDNA negativity [26, 27]. Thus, it is feasible to conduct ctDNA testing at disease progression in clinical practice to select the optimal therapeutic approach.

Our results showed a disparity in the genetic landscapes between tissue and ctDNA genotyping. Tissue-based analysis revealed a simpler *KIT* mutation status, primarily characterized by sole exon 11 mutations. In contrast, ctDNA genotyping displayed a more heterogeneous spectrum of *KIT* mutations, including the combination of *KIT* ATP-binding pocket and activation-loop mutations, reported as the predominant region of secondary mutations [9–11]. This phenomenon is likely attributable to the following factors: approximately 80% of the tissue specimens were archival samples derived from surgical procedures at the point of initial treatment, in contrast, the majority of blood samples were obtained during the phase of progressive disease or on treat after therapeutic interventions. We posit that this discrepancy underlies the observed intricate mutational status in blood samples within our investigation. Moreover, ctDNA genotyping revealed that ctDNA harboring *KIT* exon 9/11 mutations exhibited higher VAF and clonality compared with those harboring other mutations. Furthermore, ctDNA devoid of *KIT* or *PDGFR* mutations exhibited significantly diminished VAF. These observations suggest that therapeutic interventions may attenuate the prevalence of *KIT*-mutated cancer cells responsive to treatment, thereby facilitating the emergence of cells harboring mutations associated with acquired resistance. Additionally, ctDNA shedding from cells sensitive to therapeutic interventions may be less detectable when the tumor fraction is diminished by the effective treatment. Indeed, our dynamic examination of ctDNA indicates that VAF and clonality are subject to treatment-induced fluctuations and that novel mutational clones may emerge over time, as previously reported by Wada et al [28]. The phenomenon of clonal evolution occurring throughout the treatment among patients with GIST has also been posited in previous reports [29–31]. Consequently, ctDNA analysis holds potential as a tool for tracking the clonal evolution of cancer cells.

There are the distinct differences between imatinib, sunitinib, and regorafenib due to the mutational status in *KIT* exons. In alignment with previous reports, our data indicated that approximately 80% patients with ctDNA-positivity displayed resistant mutations relevant to the preceding TKIs [21, 22]. Moreover, our findings indicate the potential of ctDNA analysis to enhance therapeutic efficacy and prolong prognosis by the application of TKIs tailored to the *KIT* or *PDGFRA* mutations. While clinical guidelines, including those published by the National Comprehensive Cancer Network (NCCN) and the European Society of Medical Oncology (ESMO), provide recommendations concerning the sequence of TKIs, our results suggest the possibility of selecting therapeutic interventions on the basis of genetic mutations identified through ctDNA analysis, thereby personalizing the treatment strategy [32, 33]. While, regarding the patients with mutations for which both sunitinib and regorafenib has been considered ineffective, ripretinib, as endorsed by the NCCN and ESMO guidelines, or alternatively, the selection of pimitespib, which has garnered approval in Japan, emerge as therapeutic options according to its approval status [34, 35]. Additionally, avapritinib, identified as treatment strategy for GIST patients with *PDGFRA* D842V mutation, was recently reported to have antitumour activity in patients with a broad range of *KIT* mutations [36–38]. Furthermore, the utility of combination targeted therapy with TKIs has recently been reported [39, 40], and a phase III trial comparing combination vs. sunitinib alone is currently underway (NCT05208047). Thus, it may be possible to develop ctDNA-guided therapeutic strategies, in which single-agent or combination TKI is selected on the basis of specific mutation profiles identified through ctDNA analysis (Supplementary Fig. 5). We recognize that the categorization of ‘matched mutational status’ and ‘unmatched status’ is based on limited evidence and is not widely employed in clinical practice. Furthermore, the small sample size, encompassing various treatment lines, may introduce significant background biases, which could limit the robustness of the survival analysis. Nevertheless, our findings are supported by a recent biomarker analysis of the INTRIGUE phase III trial, published by Heinrich et al. [41]. The authors reported that ctDNA sequencing may improve the prediction of the efficacy of single-drug RTK therapies in patients with GIST. While our study provides novel insights into the potential utility of liquid biopsies in GIST management, we acknowledge the need for further validation in larger cohorts to confirm our findings and address the limitations associated with the small sample size and potential biases. Furthermore, this study also demonstrated the significant prognostic impact of ctDNA status on PFS in patients with advanced GIST. In line with research indicating ctDNA-positivity as a critical poor prognostic factor in various malignancies [42, 43], this study confirmed the profound impact of ctDNA positivity on disease trajectory and its utility as a biomarker for therapeutic decision-making in advanced GIST patients with acquired resistance to preceding TKIs.

This study has several limitations. One of the major limitations of our study is the lack of paired tissue and blood analyses for each patient. Due to the retrospective nature of our cohort and the limited availability of matched samples, we were unable to perform a comprehensive comparison of tissue and ctDNA findings at the individual patient level. This shortcoming hinders our ability to draw definitive conclusions regarding the concordance between the two approaches and may have obscured important associations between tissue and ctDNA profiles. Future prospective studies with larger cohorts and systematically collected paired samples are necessary to validate our findings and provide a more robust understanding of the relationship between tissue and ctDNA analyses in the context of GIST. Second, this study was retrospective and constrained by a limited sample size, precluding definitive conclusions. However, it constitutes an integrated analysis of four prospective trials in the SCRUM-Japan platform, and therefore, potential selection bias was mitigated. Conversely, the fact that analyses were conducted using different assay systems from four distinct trials cannot preclude the possibility that this variability might have impacted the outcomes. In sum, the sample size of this study is not sufficiently large to eliminate bias, rendering the formulation of assertive conclusions challenging. Third, because tumor size, metabolic tumor volume, or pathological characteristics, all of which are potential indicators that could influence ctDNA positivity in GIST, have not been evaluated, their impact on ctDNA positivity or *KIT* mutational status could not be assessed in this study. While we provided information on the number of metastatic organs, metastatic sites, and the presence or absence of primary tumor resection in Table 2, we did not observe any significant differences in these parameters. Future studies should aim to collect detailed information on tumor burden and investigate its association with ctDNA VAF, as this could provide valuable insights into the clinical utility of ctDNA analysis in GIST. A better understanding of the relationship between ctDNA levels and tumor volume or metabolic activity could help refine the interpretation of ctDNA results and guide treatment decision-making in patients with GIST. Finally, there was only a limited number of patients underwent longitudinal ctDNA analysis. This limitation hinders our capacity to draw robust, generalized conclusions, thus affecting the applicability of our findings. While the proportion of patients with *KIT* exon 9/11 mutations in ctDNA analysis was lower compared with previous reports, we cannot evaluate the paired pre-treatment tissue samples. *KIT* mutation at initial diagnosis was not eradicated by treatment interventions [44]. However, our findings confirmed the low maximum VAF of ctDNA without *KIT* or *PDGFRA* mutations, leading us to hypothesize that clonal evolution related to acquired resistance mechanisms may shed light on this phenomenon, potentially indicating treatment response and the low tumor fraction harboring original alterations. Additional studies are required to elucidate the influence of *KIT* mutation change or diminishing of pre-existing mutations on the sensitivity of ctDNA analyses.

In conclusion, the present study illuminated the significant correlation between ctDNA status and disease status at sampling time in advanced GIST patients and indicated ctDNA status as an independent prognostic factor. Furthermore, ctDNA-based analysis facilitated real-time evaluation of genomic profiles, including acquired mutations, emergent mutations resistant to ongoing treatments, and convergent alterations associated with malignant progression. These findings further support the utility of ctDNA in GIST, highlighting its cardinal role in therapeutic decision-making for advanced GIST patients and its potential to instigate a paradigm shift in precision medicine for this rare population, informed by molecular profiling through ctDNA testing.

## Supplementary information

Supplementary Table 1

Supplementary Table 2

Supplementary Figure 1

Supplementary Figure 2

Supplementary Figure 3

Supplementary Figure 4

Supplementary Figure 5

## Data Availability

All other data are available from the corresponding author upon reasonable request. Due to privacy laws, access to raw data is restricted. Those requests will be reviewed by a study steering committee to verify whether the request is subject to any intellectual property or confidentiality obligations.
