# Supplementary material for: Different efficacy of tyrosine kinase inhibitors by KIT and PGFRA mutations identified in circulating tumor DNA for the treatment of refractory gastrointestinal stromal tumors
Source: BJC Rep. 2024 Jul 25;2:54. doi: 10.1038/s44276-024-00073-7 (PMC11523999; doi:10.1038/s44276-024-00073-7)
Supplement: Supplementary file 1 — Supplementary Table 1 [file 44276_2024_73_MOESM1_ESM.docx]

Supplementary table 1. Cox multivariate analysis of progression-free survival

| Characteristics | | Hazard ratio (95% CI)) | *P* value |
| --- | --- | --- | --- |
| Age (years) | |  |  |
|  | ≤65 | 1 | 0.948 |
|  | >65 | 0.97 (0.36-2.61) |  |
| Sex | |  |  |
|  | Male | 1 | 0.250 |
|  | Female | 0.57 (0.22-1.48) |  |
| Tumor location | |  |  |
|  | Stomach | 1 | 0.623 |
|  | Other than stomach | 1.27 (0.50-3.24) |  |
| Resection of primary tumor | |  |  |
|  | Performed | 1 | 0.315 |
|  | Not performed | 1.49 (0.68-3.27) |  |
| Number of metastatic organ sites | |  |  |
|  | ≤1 | 1 | 0.671 |
|  | ≥2 | 1.24 (0.23-2.79) |  |
| Number of previous treatment regimens | |  |  |
|  | ≤2 | 1 | 0.132 |
|  | ≥3 | 2.46 (0.74-8.22) |  |
| ctDNA status | |  |  |
|  | Negative | 1 | 0.038 |
|  | Positive | 3.46 (1.07-11.2) |  |

Abbreviations: ctDNA, circulating tumor DNA; 95% CI, 95% confidence interval.
