# Supplementary material for: Different efficacy of tyrosine kinase inhibitors by KIT and PGFRA mutations identified in circulating tumor DNA for the treatment of refractory gastrointestinal stromal tumors
Source: BJC Rep. 2024 Jul 25;2:54. doi: 10.1038/s44276-024-00073-7 (PMC11523999; doi:10.1038/s44276-024-00073-7)
Supplement: Supplementary file 2 — Supplementary Table 2 [file 44276_2024_73_MOESM2_ESM.docx]

Supplementary table 2. Pathogenic mutational dynamics of ctDNA analysis in patients with multiple blood sampling

| Patients | First analysis | | Second analysis | |
| --- | --- | --- | --- | --- |
|  | Mutations | VAF | Mutations | VAF |
| #1 | *KIT* 1648-9_1672del34  *DNMT3A* 1226_1227insTG  *NKX2-1* amp | 8.2  2.13  CN, 15 | *KIT* 1648-9_1672del34  *KIT* V654A  *DNMT3A* 1226_1227insTG  *DNMT3A* 958C>T  *ATR* 2320_2321insA | 0.7  0.44  3.62  0.7  4.84 |
| #2 | *PDGFRA* D842_S847>EF | 3.47 | *PDGFRA* D842_S847>EF  *CDKN2A* rearrangement exon 2  *MTAP* rearrangement intron 6 | 5.02 |
| #3 | *KEL* L505fs*5  *SGK1* F430fs*6 | 0.24  1.6 | *SGK1* F430fs*6 | 1.18 |

Abbreviations: VAF, variant allele frequency; amp, amplification.
