# Supplementary figures and images for: Different efficacy of tyrosine kinase inhibitors by KIT and PGFRA mutations identified in circulating tumor DNA for the treatment of refractory gastrointestinal stromal tumors
Source: BJC Rep. 2024 Jul 25;2:54. doi: 10.1038/s44276-024-00073-7 (PMC11523999; doi:10.1038/s44276-024-00073-7)

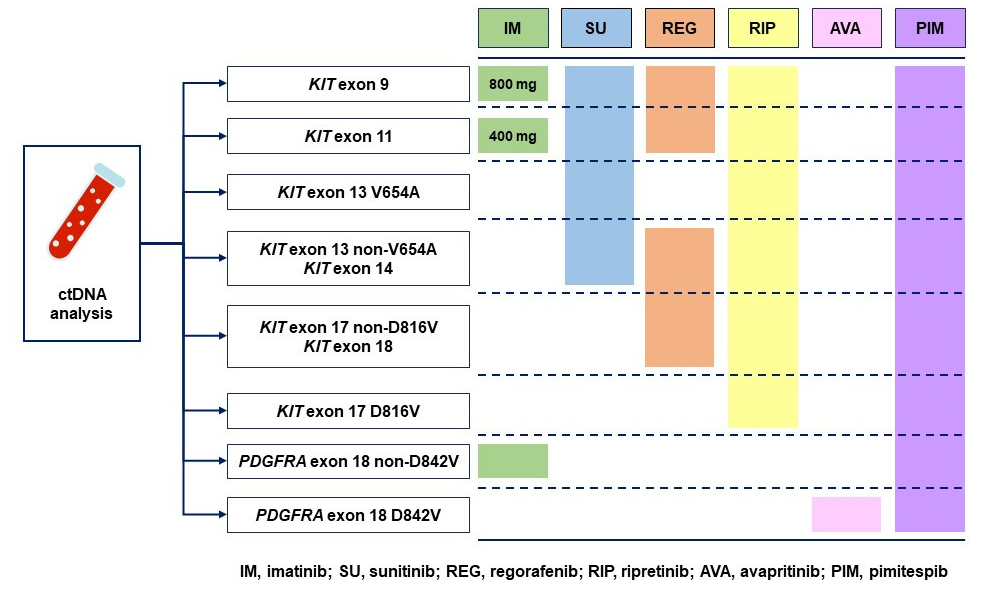

Supplement: Supplementary file 3 — Supplementary Figure 1 [file 44276_2024_73_MOESM3_ESM.jpeg]

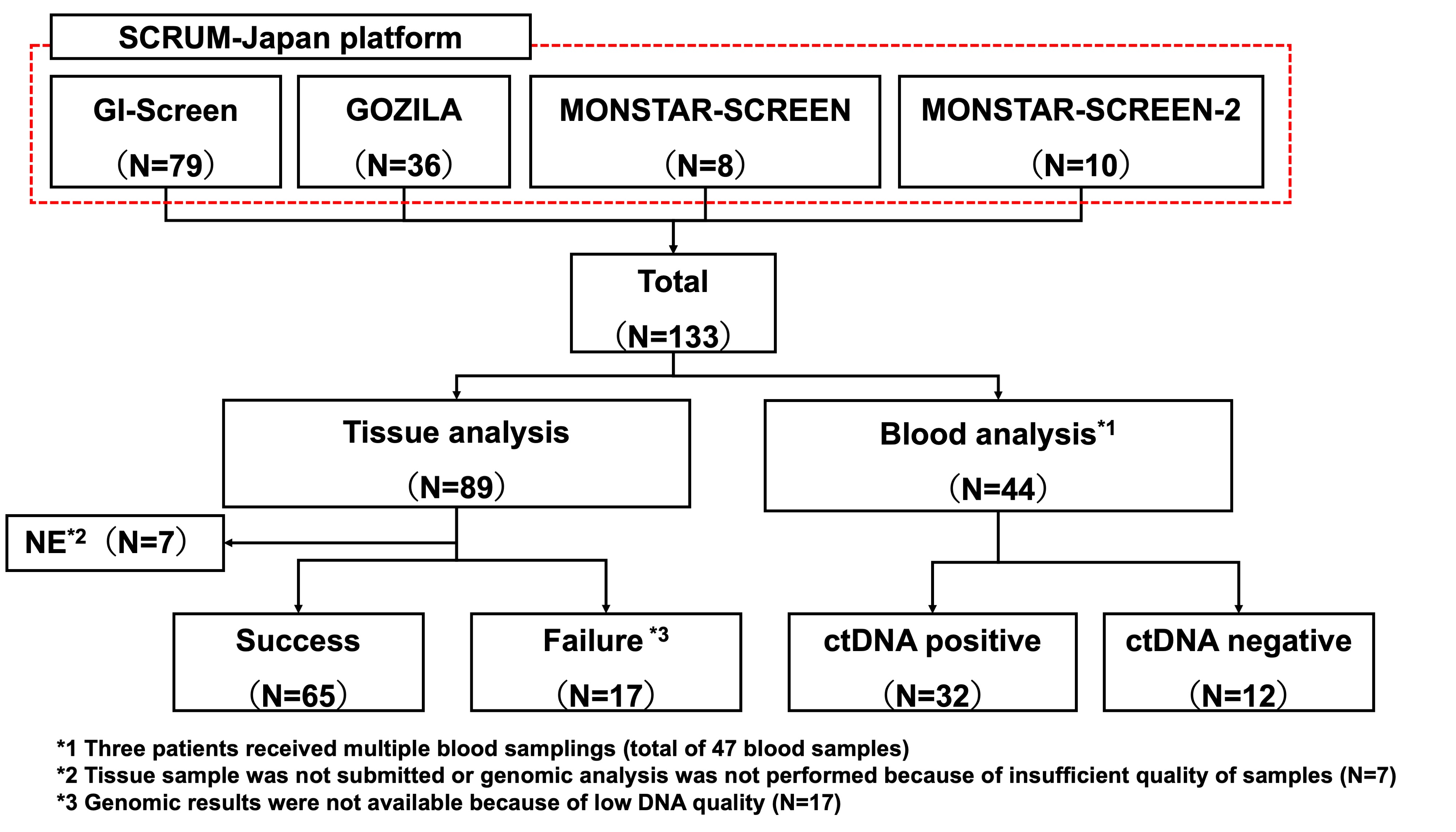

Supplement: Supplementary file 4 — Supplementary Figure 2 [file 44276_2024_73_MOESM4_ESM.jpg]

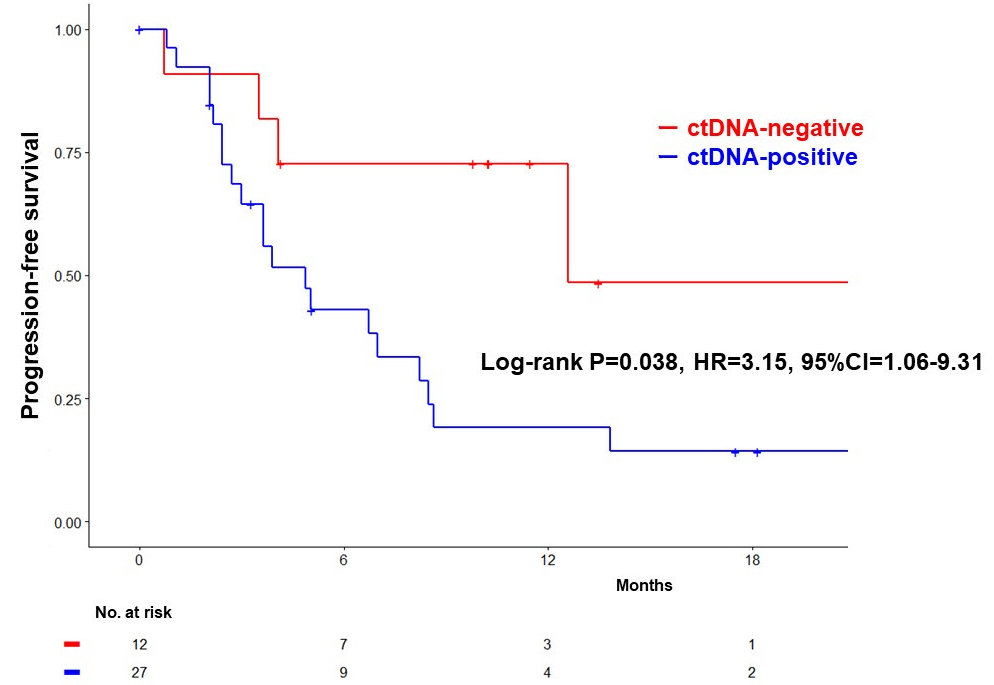

Supplement: Supplementary file 5 — Supplementary Figure 3 [file 44276_2024_73_MOESM5_ESM.jpeg]

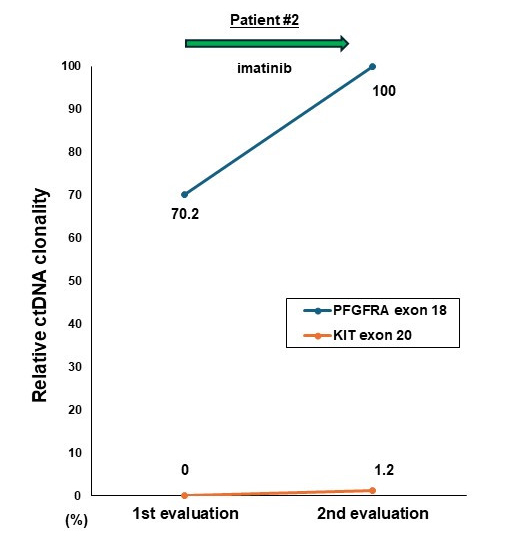

Supplement: Supplementary file 6 — Supplementary Figure 4 [file 44276_2024_73_MOESM6_ESM.jpeg]

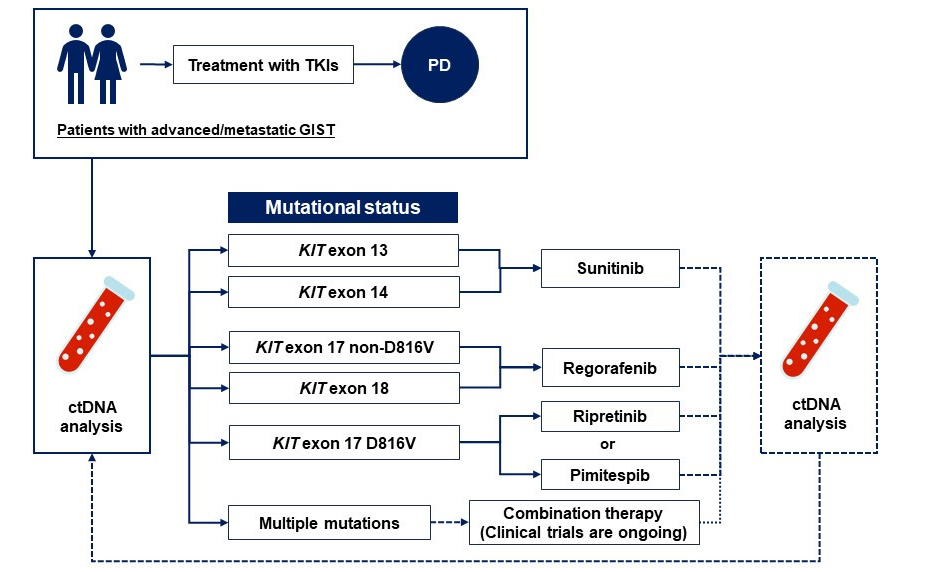

Supplement: Supplementary file 7 — Supplementary Figure 5 [file 44276_2024_73_MOESM7_ESM.jpeg]
